# Changes in Ibuprofen Toxicity and Degradation in Response to Immobilization of *Bacillus thuringiensis* B1(2015b)

**DOI:** 10.3390/molecules29235680

**Published:** 2024-11-30

**Authors:** Ariel Marchlewicz, Anna Dzionek, Danuta Wojcieszyńska, Jacek Borgulat, Łukasz Jałowiecki, Urszula Guzik

**Affiliations:** 1 Institute of Biology, Biotechnology and Environmental Protection, Faculty of Natural Science, University of Silesia in Katowice, Jagiellońska 28, 40-032 Katowice, Poland; ariel.marchlewicz@us.edu.pl (A.M.); anna.dzionek@us.edu.pl (A.D.); danuta.wojcieszynska@us.edu.pl (D.W.); 2 Institute for Ecology of Industrial Areas, Kossutha 6, 40-844 Katowice, Poland; j.borgulat@ietu.pl (J.B.); l.jalowiecki@ietu.pl (Ł.J.)

**Keywords:** kinetics, immobilization, toxicity, ibuprofen, *Bacillus*

## Abstract

Ibuprofen is one of the most commonly used anti-inflammatory drugs by humans, resulting in its appearance in the environment, which can negatively affect organisms living in it. The studies undertaken have shown that the immobilized *Bacillus thuringiensis* B1(2015b) strain can decompose this drug at a rate of *q_max_* = 0.36 mg/L*h, with a *K_s_* constant of 0.95 mg/L for this process. An analysis of the effect of ibuprofen on the metabolic profile of the immobilized strain B1(2015b) showed an increase in the consumption of carbon, nitrogen, phosphorus, and sulfur compounds by this strain compared to the free strain. Studies on the toxicity of ibuprofen against the B1(2015b) strain indicated a small protective effect of the carrier, manifested by a slightly higher EC_50_ value = 1190 mg/L (for the free strain EC_50_ = 1175 mg/L). A toxicity analysis of intermedia formed during ibuprofen degradation indicated that the increase in toxicity is positively correlated with the degree of hydroxylation of ibuprofen metabolites. A toxicity analysis of the post-culture fluid obtained after ibuprofen degradation by the immobilized and free strain indicated that the products formed due to this process are completely safe.

## 1. Introduction

Ibuprofen is one of the most commonly used analgesics and anti-inflammatory drugs. Its mechanism of action is based on the non-specific inhibition of cyclooxygenases responsible for synthesizing prostaglandins from arachidonic acid [1,2]. It is not entirely metabolized in the human body, transforming into phase I and II detoxification products, such as carboxy-ibuprofen, hydroxylated ibuprofen derivatives, and their glucuronic and sulfate derivatives [3]. These metabolites are excreted from the body in 85% of urine and feces and end up in sewage [4,5]. In the aquatic environment, metabolites are most often hydrolyzed to the parent product or its hydroxyl derivatives [6]. An analysis of these compounds in sewage treatment plants has shown that they occur in the concentration range from ng/L to µg/L [3,5]. The incomplete degradation of these compounds in sewage treatment plants leads to their appearance in the environment, which may affect the organisms in it [4,7].

The presence of the methyl propyl group in the structure of ibuprofen gives it a lipophilic character (log K_ow_ = 3.49). For this reason, this drug is characterized by the ability to penetrate biological membranes and distribute in living organisms [8]. Studies have shown the bioaccumulation of ibuprofen in living organisms such as *Mytilus trossulus* and *Mytilus galloprovincialis* [9]. This accumulation, in turn, can lead to adverse effects, such as oxidative stress, DNA damage, reduced enzymatic activity, and mitochondrial damage [8,10,11]. Minimizing ibuprofen concentrations in sewage treatment plant effluents is crucial to reducing the risk of its harmful effects on living organisms. For this purpose, biological oxidation methods using strains with increased degradation potential are increasingly used [12]. For decades, bacteria belonging to genera such as *Nocardia*, * Sphingomonas*, * Cycloclasticus*, * Variovorax*, *Citrobacter*, or *Patulibacter* have been described as demonstrating the ability to degrade ibuprofen. The advantage of using microorganisms to degrade ibuprofen is primarily the complete mineralization of this drug, which avoids the accumulation of toxic intermediates observed during biotransformation reactions or physicochemical processes. Biological processes are environmentally friendly and economically viable. However, their disadvantages are low efficiency and the rare possibility of using laboratory strains in technological conditions due to their low survival [10]. The solution to the problem of low survival of selected strains may be their immobilization. Previous studies indicate that immobilized strains are characterized by lower sensitivity to changing environmental conditions and competition from the autochthonous microbiome of the environment [13]. However, immobilization can also change their biological activity, which affects the efficiency of the conducted processes and the formation of degradation intermediates [14]. In the current work, the authors used a well-described strain of *Bacillus thuringiensis* B1(2015b) to degrade ibuprofen to check how immobilization will affect the intensity of the degradation process of this drug. In addition, an attempt was made to determine the toxicity of ibuprofen and its primary metabolites to the free and immobilized strain B1(2015b). An essential aspect of the work was also to determine whether the post-culture fluid obtained as a result of the decomposition process by the immobilized strain may have a negative impact on aquatic organisms. Such studies allowed us to answer the question of whether the use of the immobilized strain is associated with an increased environmental risk.

## 2. Results and Discussion

The *B. thuringiensis* B1(2015b) strain has been described as degrading ibuprofen with high efficiency [15]. Due to the application potential of the strain in bioremediation of ibuprofen-contaminated environments, its immobilization on carriers is necessary. Previous studies have shown that this strain can degrade nonsteroidal anti-inflammatory drugs after immobilization [16]. However, no comprehensive studies have been conducted to date on how immobilization affects the metabolic activity of the B1(2015b) strain and the kinetics of ibuprofen degradation.

### 2.1. Kinetic Degradation by Free and Immobilized Bacteria

In the first stage of the study, the kinetics of ibuprofen degradation by the free and immobilized B1(2015b) strain on a plant sponge were compared.

**Figure 1:**
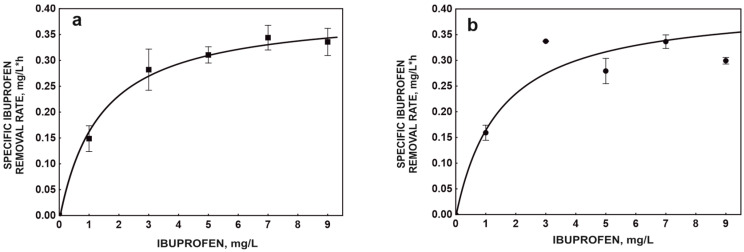
Kinetic models of ibuprofen degradation by free (**a**) and immobilized (**b**) cells of *Bacillus thuringiensis* B1(2015b). The data points represent the average of three independent experiments.

An interesting result is the kinetic parameters determined for the free strain. Maximum specificity ibuprofen removal rate—*q_max_* is 0.41 mg/L*h, while half-saturation constant—*K_s_* is 1.51 mg/L (Figure 1a). Compared to the parameters obtained earlier (*q_max_* = 0.24 mg/L*h and *K_s_* = 2.12 mg/L) by Marchlewicz et al. [15], the currently obtained kinetic constants indicate an improvement in the degradation properties of the strain, which is probably due to the long-term exposure of the strain to ibuprofen and increased induction of degradative enzymes. After immobilization, the B1(2015b) strain degraded ibuprofen at a rate of *q_max_* = 0.36 mg/L*h, and the *K_s_* value was 0.95 mg/L (Figure 1b).

**Figure 2:**
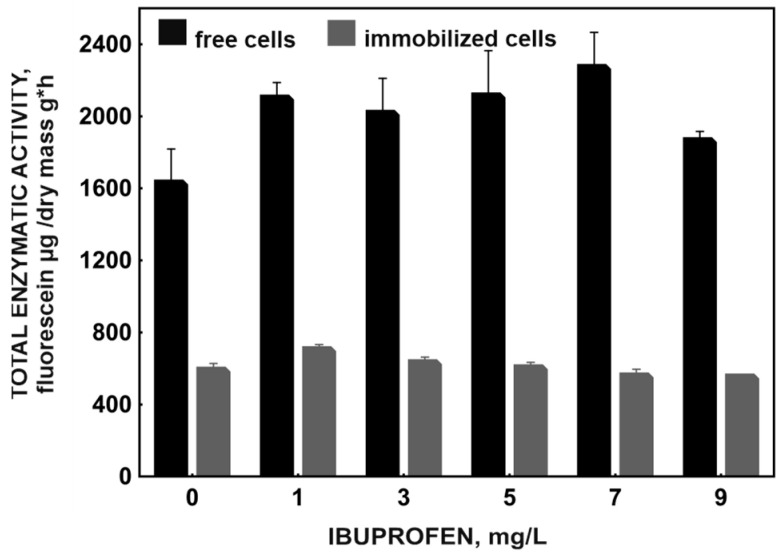
Changes in total enzymatic activity during degradation of different ibuprofen concentrations by free and immobilized cells of *Bacillus thuringiensis* B1(2015b). All experiments were performed in at least three replicates.

The observed values indicate a slight decrease in the rate of ibuprofen degradation after strain immobilization, which is also reflected in the reduced total metabolic activity of the strain (Figure 2). Although immobilization slightly reduced the degradation potential of the B1(2015b) strain, the undoubted advantage of using the carrier is the possibility of reusing the preparation constructed in this way. In addition, the immobilized strain is more resistant to changing environmental conditions and competition from the autochthonous microbiome of activated sludge [13].

The immobilization method used is based on the ability to form a biofilm on the carrier [16]. Although microorganisms of the compact biofilm structure strive to create structural integrity and stability, it is often observed that this structure can cause a reduction in metabolic activity [17]. This decrease is partly due to the fact that intermediates formed due to degradation do not leave the reaction site, accumulating in specific domains of the biofilm and thus affecting bacterial cells [13,18].

Strain B1(2015b) degrades ibuprofen via 2-hydroxyibuprofen, for which aliphatic monooxygenase is responsible. The obtained intermediate is characterized by high stability. Hence, it is a factor that limits the degradation of ibuprofen by this strain. Further degradation of this intermediate occurs through its transformation into 2-(4-hydroxyphenyl)-propionic acid, which in turn is transformed into 1,4-hydroxyquinone, which is hydroxylated to benzenetriol. The latter is cleaved by 1,2-hydroxyquinol dioxygenase to an aliphatic product incorporated in the central metabolism [19]. Hydroxylated metabolites that are more toxic than ibuprofen can negatively affect the metabolic activity of B1(2015b) strain cells [10]. Additionally, such a structure can constitute a diffusion barrier, reducing the concentration of the degraded drug in the microenvironment of the carrier [13]. Perhaps the adaptive increase in the affinity of degradative enzymes, as evidenced by the reduced K_s_ value, for the substrate is the strain’s response to a low drug concentration.

### 2.2. Analysis of the Effect of Ibuprofen on the Metabolic Profile of B1(2015b) Strain

The demonstration of differences in the metabolic activity of free and immobilized cells in the ibuprofen presence indicated the need to undertake analyses of the metabolic profile of free and immobilized cells to demonstrate the differences between them. A helpful and efficient tool for achieving this goal is phenotypic microarrays (PMs), which are used to study microorganisms’ metabolic and stress responses. These microplates contain dried chemicals that, when hydrated, create different culture conditions. They reveal which metabolic pathways are active and which are inhibited. They are divided into functional groups such as C, N, P, and S metabolism, ion sensitivity, osmotic effects, pH, and chemicals [18].

**Figure 3:**
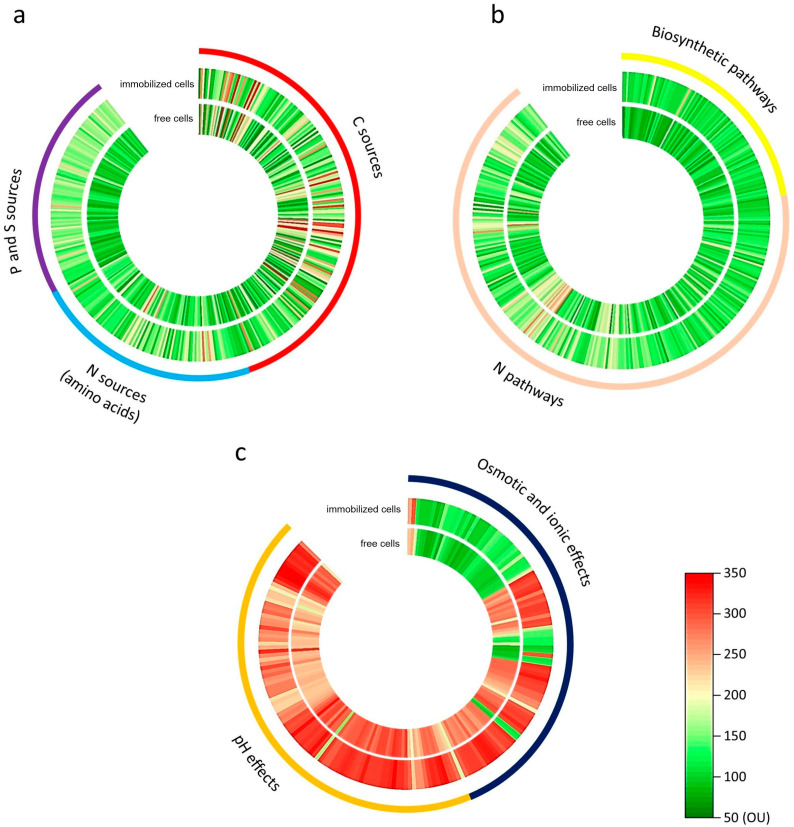
Microarray phenotyping (PM) analysis results obtained during 5 mg/L ibuprofen exposition of free and immobilized *Bacillus thuringiensis* B1(2015b). The microplates in the PM have been divided into seven functional groups: (**a**)—carbon sources, nitrogen sources, sulfur, and phosphorus sources; (**b**)—biosynthetic pathways and nitrogen pathways; (**c**)—osmotic and ion effects and pH effects. OU—OmniLog units.

As a result of analyses of all functional groups, statistically significant differences were shown (Table 1) within the analyzed functional groups between the free and immobilized B1(2015b) strain after exposure to 5 mg/L ibuprofen (Figure 3). This analysis indicated an increased use of carbon, nitrogen, phosphorus, and sulfur sources by immobilized cells, which was also reflected in the intensity of biosynthetic and nitrogen metabolism processes.

Pronounced changes were observed in the metabolism of nitrogen, phosphorus, and sulfur compounds, potentially linked to the enhanced synthesis of small peptides that are quorum-sensing signaling molecules. They play a considerable role, among others, in forming biofilm and regulating the population forming it, especially under stressful conditions such as the presence of ibuprofen [20,21]. The synthesis processes of these small peptides are necessary for the proper functioning of the biofilm. Still, their presence does not determine the increase in the overall activity of the cells. This can explain the low total metabolic activity of the B1(2015b) strain (Figure 2) after immobilization despite the increase in the metabolic profile parameters (Figure 3). Confirmation of the relationship between the increased metabolism of nitrogen, phosphorus, and sulfur compounds and the synthesis of small signal peptides requires further detailed studies.

The resistance of bacteria in bioremediation to changing environmental conditions is extremely important. An analysis of Biolog microplates (9–10 PM) indicated an increased ability to grow immobilized bacteria in the presence of osmolar compounds and a more comprehensive range of pH (Table 1, Figure 3c). This adaptability enables their use in ibuprofen degradation in a broader range of environmental changes.

### 2.3. Impact of Immobilization on the Changes in Intermediate Toxicity During Ibuprofen Degradation

Ibuprofen, although broken down by the B1(2015b) strain, is not a neutral substance for it. Marchlewicz et al. [15] showed that in the presence of 809 mg/L of this drug, there is a 50% inhibition of the growth of the B1(2015b) strain. They showed that ibuprofen has a high affinity for the lipid bilayer due to its amphipathic structure. In response to this drug, the B1(2015b) strain changes its membrane fatty acid composition towards an increase in the amount of branched fatty acids together with the high content of long-chain fatty acids relative to unsaturated fatty acids. This modification modulates the fluidity of cell membranes by increasing the phase transition temperature, which leads to a decrease in membrane permeability [15].

The studies showed minor changes in the sensitivity of the immobilized B1(2015b) strain to ibuprofen compared to the free strain. The EC_50_ for the free strain was determined to be 1175 mg/L, a higher value than the EC_50_ value (809.3 mg/L) previously defined for this strain by Marchlewicz et al. [15]. This suggests that long-term passage of the strain in the presence of ibuprofen has fixed the features that determine its resistance to this drug. The EC_50_ value increased slightly for the immobilized strain, similar to the non-inhibitory concentration (NIC) value (Table 2).

This indicates that immobilization may slightly affect the protection of the strain against the toxic effects of ibuprofen. Surprisingly, however, the minimum inhibitory concentration (MIC) value is lower for the immobilized strain than for the free strain. This may suggest that after exceeding a particular threshold value, the carrier no longer plays a protective role, while the structure of the biofilm is subject to less dynamic changes than free cells. The documented negative effect of ibuprofen on membranes may cause a change in the permeability of bacterial cell membranes, which may consequently lead to the loosening of mature biofilm. Such an effect was observed, among others, in the case of subtilins produced by *Staphylococcus simulans*, which induce the dispersion of transmembrane electrostatic potentials, consequently disrupting the biofilm structure [22]. Studies have shown that ibuprofen can inhibit some signaling pathways through its structural similarity to other signaling molecules or inhibitors. One of the known inhibitors of transcription of the *relA* gene encoding (p)ppGpp-synthetase/hydrolase is eugenol. The inhibition of the synthesis of this enzyme reduces the pool of alarmones, which consequently disrupts biofilm formation. Ibuprofen is structurally similar to eugenol and may have similar effects. The disruption of the biofilm structure may result in greater strain sensitivity to this drug [23]. The described effects may cause the lower MIC value observed for the immobilized strain (Table 2).

The analysis of the toxicity of intermediates formed during the degradation of ibuprofen indicated that their toxicity increases with the degree of hydroxylation according to the series 1-hydroxyibuprofen<catechol<1,2,4-benzenetriol (Table 2). This confirms previous reports that hydroxylation increases toxicity [24,25]. Surprisingly, no protective effect of the carrier was observed, resulting in the immobilized strain being more sensitive to metabolites than the free strain. The analysis of the toxicity of the ibuprofen intermediates, and the post-culture fluid indicated a significant decrease in toxicity, which is associated with the effective degradation of ibuprofen by both the free and immobilized strain B1(2015b). The analysis of the toxicity of ibuprofen and the post-culture fluids using shrimp as model organisms also indicated the lack of toxicity of the drug and the post-culture fluids (Table 3). The Shapiro–Wilk test confirmed a lack of normality in all groups (*p* < 0.05). The Kolmogorov–Smirnov test revealed no statistically significant differences between concentration groups, with *p*-values exceeding 0.05 in all comparisons. The results showed that ibuprofen degradation by both free and immobilized B1(2015b) strains led to safe degradation products.

## 3. Materials and Methods

### 3.1. Immobilization of B. thuringiensis B1(2015b) Strain

*B. thuringiensis* B1(2015b) strain (GeneBank accession number KP895873.1), isolated from the soil of the chemical factory Organika-Azot in Jaworzno, Poland [15], was immobilized through the adsorption on the surface of the loofah sponge according to Dzionek et al. [16]. The prepared preparation was used in further experiments.

### 3.2. Biochemical Analysis

#### 3.2.1. Assay of Total Enzymatic Activity Free and Immobilized Bacteria

The metabolic activity of free and immobilized microorganisms was measured using the fluorescein diacetate method (FDA), according to Dzionek et al. [16,26]. This method is based on FDA hydrolyzation using non-specific extracellular and membrane-bound enzymes. The measurement of enzyme activity using FDA correlates with parameters such as biomass, ATP content, oxygen consumption, or optical density and is therefore used to express total enzymatic activity [26].

#### 3.2.2. Determination of Ibuprofen Concentration

In order to determine ibuprofen concentration, medium samples from each flask were taken and centrifuged (14,000 rpm, 20 min). The concentration of ibuprofen in the supernatant was evaluated through RP-HPLC (Merck HITACHI, Darmstadt, Germany) equipped with an Ascentis Express ^®^ C18 HPLC Column (100 × 4.6 mm), an Opti-Solw ^®^ EXP pre-column and a DAD detector. The mobile phase consisted of acetonitrile and 1% acetic acid (70:30 *v*/*v*) at a 1 mL/min flow rate. The detection wavelength was set at 240 nm [15]. Ibuprofen was identified by comparison of HPLC retention time (2.77 ± 0.03 min) and UV-VIS spectra with those from the external standards.

#### 3.2.3. Metabolic Profile Analysis

Metabolism of various carbon and nitrogen sources and response to various pH values for free and immobilized *B. thuringiensis* B1(2015b) strain growing in the presence of ibuprofen was evaluated by using BIOLOG (Hayward, USA) phenotype microarrays (PM 01, PM 02, PM 03, PM 04, PM 05, PM 09, and PM 10). Free cells of the B1(2015b) strain were propagated in an LB medium with 5 mg/L ibuprofen. Then, the bacterial biomass was centrifuged (15 min/5000 rpm/4 °C), transferred to mineral salt (MS) medium [15] with 5 mg/L ibuprofen, and incubated for 14 days. The cultures were supplemented with glucose at 0.5 g/L at 48 h intervals. The cultures were passaged after reaching an optical density (OD_600_) of 1.0–1.2. A suspension of bacteria from a 24 h culture was applied to PM plates. The strain immobilized on a loofah sponge, according to Dzionek et al. [16], was released from the carrier. Briefly, cells were released from the carrier by shaking and suspended in NaCl solution (0.9%), centrifuged (14,000 rpm, 20 min), and resuspended in physiological saline. The suspensions of B1(2015b) cells prepared this way were introduced into 1x IF-0a to obtain a transmittance of 81% and used to prepare the 1xPM inoculating solution according to the manufacturer’s instructions. Then, inoculates were transferred to the appropriate PM1–10 plates and incubated using the Omnilog Incubator/Reader (Biolog Inc., Hayward, CA, USA) for 7 days. PM 03 and PM 10 microarrays were additionally supplemented with 20 mM of pyruvate. The changes of color in the wells, which are the results of tetrazolium color formation, were measured every 15 min. Analysis of the color development as a function of time was performed using OmniLog^®^ MicroArray Data Collection Software Release 1.2 (Biolog Inc, USA.) [27]. Data visualization was performed using a DuctApe program [28].

### 3.3. Ibuprofen Degradation Kinetics Study

To study degradation kinetics, ibuprofen decomposition by B1(2015b) strain was monitored in a 500 mL Erlenmeyer flask containing 250 mL of the mineral salt medium [29]. Each flask was supplemented with ibuprofen at initial concentrations of 1, 3, 5, 7, and 9 mg/L of bacterial inoculum to obtain an initial OD_600_ equal to 0.1 and glucose (0.5 g/L). Samples were taken every 2 h to determine ibuprofen concentration and monitor the growth of the culture by spectroscopic measurement of the optical density at 600 nm.

Kinetic constants were determined using GraphPad Prism 8 software based on the following Monod Equation (1):

(1) $$q=\frac{q_{max}\times S}{K_{S}+S}$$

where *q* is the specific ibuprofen removal rate (mg/L*h), *q_max_* is the maximum specific ibuprofen removal rate (mg/L*h), *K_s_* is the half-saturation constant (mg/L), and *S* is the ibuprofen concentration (mg/L) [15].

### 3.4. Toxicity Study

#### 3.4.1. Ibuprofen and Its Metabolite Toxicity Against to B1(2015b) Strain

*B. thuringiensis* B1(2015b) was routinely cultivated in the nutrient broth (BD Life Sciences, Becton, Franklin Lakes, NJ, USA) at 30 °C and 130 rpm for 24h [15]. After this, cells were harvested by centrifugation (5000× *g* at 4 °C for 15 min), washed with a fresh sterile mineral salts medium [29], and used as inoculum.

The strain was immobilized on a loofah sponge and subsequently released from the carrier as outlined by Dzionek et al. [16].

The toxicity assay of ibuprofen and its metabolites was conducted based on bacterial growth inhibition and OD_600_ measurements. The tests were carried out on 96-well microtiter plates. To determine the toxicity of ibuprofen and its metabolites—catechol, 1-hydroxyibuprofen, 1,2,4-benzenetriol, and 2-(4-hydroxyphenyl)propionic acid, serial dilutions (with the highest concentration of 2002.5 mg/L and a dilution factor of 1.5) of the selected compounds were prepared. The choice of metabolites resulted from previously conducted studies on the metabolic pathway of ibuprofen in the B1(2015b) strain, where they were identified [19]. All chemicals were suspended in LB broth and sterilized using a 0.22 µm cellulose syringe filter. For dilutions, sterile LB broth was used.

To determine the toxicity of post-culture medium concentrations of 100%, 50%, 20%, and 10% on the microtiter plates were prepared. The bacterial cells from the free and immobilized system were suspended in sterile LB broth and used for inoculation in the growth inhibition test.

In the first step, the wells were filled with 180 µL of the prepared dilutions, and then 20 µL of the bacterial suspension was added to achieve an initial optical density of 0.05. Optical density measurements of the cultures were performed using a Tecan Spark 10 multi-plate reader (Tecan Trading AG, Männedorf, Switzerland). The measurements were conducted in quadruplication; the 96-well plates were sealed with Parafilm, covered, and incubated at 30 °C with shaking at 110 RPM for 24 h. EC_50_, MIC, and NIC values were estimated using GraphPad Prism 8.4.3 software using nonlinear regression (EC_50_ shift) and Gompertz equation for MIC and NIC determination.

#### 3.4.2. Test Thamnotox

The acute toxicity assessment using the Thamnotox assay was performed according to the standard procedures outlined by the manufacturer. The Thamnotoxkit (Microbiotests Inc., Gent, Belgium) is a 24 h acute toxicity test utilizing fairy shrimp larvae *Thamnocephalus platyurus* hatched from cysts.

The hatching of cysts was initiated 16–18 h before the onset of toxicity testing. The cysts were rehydrated for 1 h before being transferred to a Petri dish containing standard hatching and dilution medium (EPA hard water). The inactive eggs were then incubated at 25 °C under continuous illumination at approximately 2800 lux. The larvae were ready for testing 16–18 h post-incubation. The ibuprofen or post-culture media dilution series was prepared in EPA hard water. Both pure standard compound and post-culture media were analyzed to assess any potential increase in toxicity following degradation. The concentration levels of pure compounds were matched to those in the post-culture medium to enable toxicity comparison. The highest concentration of ibuprofen was 10 mg/L, with serial dilutions down to a minimum of 0.313 mg/L (with a dilution factor equal to 2). Post-culture media were used from ‘free’ bacteria cultures and immobilized cultures with dilution rates of 100, 50, 25, 12.5, 6.25, and 3.125% compared to the control (fresh hard water).

The wells of the test plates were filled with 1 mL of standard EPA hard water as a control, along with each of the corresponding toxicant concentrations. Ten larvae were transferred into each well from the Petri dish (30 individuals per concentration). Each toxicant concentration was tested in three replicate wells. A strip of Parafilm was placed on top of the multiwell plate before covering it. The test plates were then incubated at 25 °C in darkness for 24 h. The number of dead and living larvae in each test well was recorded to assess the results. The mean mortality percentage was calculated for the three replicates, and the 24 h LC_50_ was estimated using graphical interpolation on the calculation sheet provided by the manufacturer.

### 3.5. Statistical Analysis

All experiments were performed in at least three replicates. Mean values and statistical errors were computed. All results underwent statistical analysis using Statistica 13.0 software. A statistical analysis was performed to evaluate the significance of differences in survival rates across ibuprofen concentrations in different systems (0–10 mg/L). First, the Shapiro–Wilk test was applied to assess the normality of data within each concentration group. In all cases, the *p*-value was below 0.05, indicating a rejection of the null hypothesis of normal distribution. Subsequently, the Kolmogorov–Smirnov test was used to compare the distributions between groups (concentrations).

## 4. Conclusions

In summary, the results show that although immobilization slightly reduces the rate of ibuprofen degradation by the B1(2015b) strain and reduces its total metabolic activity, binding the strain to the carrier protects this strain not only from the toxic effects of ibuprofen itself but also from its hydroxylated metabolites. Furthermore, the results confirmed that ibuprofen degradation by this strain leads to safe degradation products, allowing its effective use in biotechnological processes.

## Figures and Tables

**Table 1:** Metabolic and stress response of free and immobilized *Bacillus thuringiensis* B1(2015b) in the presence of ibuprofen obtained as a microarray phenotyping analysis.

Functional Group	Free Cells *	Immobilized Cells *
C sources	121 ± 52.4 ^a^	147 ± 52.2 ^b^
N sources	108 ± 29.7 ^a^	127 ± 37.9 ^b^
S and P sources	94 ± 14.2 ^a^	138 ± 18.8 ^b^
Biosynthetic pathways	94 ± 14.8 ^a^	107 ± 15.9 ^b^
N pathways	121 ± 30.2 ^a^	138 ± 42.4 ^b^
Osmotic and ion effects	193 ± 78.9 ^a^	216 ± 91.4 ^b^
pH effects	258 ± 35.5 ^a^	286 ± 40.2 ^b^

* Different letters indicate significant differences (*p* < 0.05, LSD test) related to the effects of ibuprofen on bacterial strain.

**Table 2:** Toxicity parameters of ibuprofen and its metabolites estimated towards free and immobilized *Bacillus thuringiensis* B1(2015b).

	Free Cells			Immobilized Cells		
Compounds	EC_50_ mg/L	MIC mg/L	NIC mg/L	EC_50_ mg/L	MIC mg/L	NIC mg/L
Ibuprofen	1175.0	1909.0	533.0	1190.0	1798.0	603.7
Catechol	36.9	67.2	21.7	39.5	62.2	26.4
1-hydroxyibuprofen	809.3	1103.0	577.2	787.5	1102.0	547.1
1,2,4-benzenetriol	35.5	371.7	3.6	27.5	60.0	13.2
2-(4-hydroxyphenyl)propionic acid	Lack of toxicity to 2 g/L			Lack of toxicity to 2 g/L		

**Table 3:** The survival rate in percentage of crustacean, *Thamnocephalus platyurus*, in THAMNOTOXKIT F toxicity test. Results are shown for three experimental setups: (1) containing ibuprofen, (2) post-culture broth from a degradation system with free bacteria, and (3) post-culture broth from a degradation system with bacteria immobilized on loofah sponges.

	**Concentration [mg/L]**					
	0	0.625	1.25	2.50	5.00	10.00
	**Survival rate [%]** *					
**Ibuprofen**	100.0 ^a^ ± 0.0	100.0 ^a^ ± 0.0	100.0 ^a^ ± 0.0	100.0 ^a^ ± 0.0	100.0 ^a^ ± 0.0	96.7 ^a^ ± 0.0
	**Concentration [%]**					
	0	0.625	12.50	25.00	50.00	100.00
	**Survival rate [%]** *					
**Ibuprofen’s post-breeding broth (free cells system)**	100.0 ± 0.0 ^a^	100.0 ± 0.0 ^a^	100.0 ± 0.0 ^a^	100.0 ± 0.0 ^a^	96.7 ± 0.0 ^a^	86.7 ± 0.0 ^a^
**Ibuprofen’s post-breeding broth (immobilized cells system) [%]**	100.0 ± 0.0 ^a^	100.0 ± 0.0 ^a^	100.0 ± 0.0 ^a^	100.0 ± 0.0 ^a^	100.0 ± 0.0 ^a^	90.0 ± 10.0 ^a^

* Mean values followed by the same letter are not significantly different according to Kolmogorov–Smirnov test (*p* > 0.05). Different letters indicate statistically significant differences (*p* < 0.05). Statistical tests were performed on each system.

## Data Availability

The data presented in this study are available on request from the corresponding author.

## References

[1] Huang C.Y., Fu L.H., Sung M.H., Huang C.F., Wu J.P., Kuo H.W. (2020). Ibuprofen biodegradation by hospital, municipal, and distillery activated sludges. Environ. Technol..

[2] Ogunwole G.A., Saliu J.K. (2020). Seasonal occurence of ibuprofen in sediment, water, and biota in river Owena and Ogbese, and its ecological risk assessment. Ann. Sci. Technol..

[3] Salgado R., Brito D., Noronha J.P., Almeida B., Bronze M.R., Oehmen A., Carvalho G., Crespo M.T.B. (2020). Metabolite identification of ibuprofen biodegradation by *Patulibacter medicamentivorans* under aerobic conditions. Environ. Technol..

[4] Aulestia M., Flores A., Acosta-Jurado S., Santero E., Camacho E.M. (2022). Genetic characterization of the ibuprofen-degradative pathway of *Rhizorhabdus wittichii* MPO218. Appl. Environ. Micorbiol..

[5] Özgüven A., Öztürk D., Bayram T. (2021). An investigation based on removal of ibuprofen and its transformation products by a batch activated sludge procesess: A kinetic study. Environ. Res. Tec..

[6] Ossowicz-Rupniewska P.E., Kucharska E., Klebeko J., Kopciuch E., Bilska K., Janus E. (2023). Effect of the type of amino acid on the biodegradation of ibuprofen derivatives. Arch. Environ. Protect..

[7] Balciunas E.M., Kappelmeyer U., Harms H., Heipieper H.J. (2020). Increasing ibuprofen degradation in constructed wetlands by bioaugmentation with gravel containing biofilms od an ibuprofen-degrading *Sphingobium yanoikuyae*. Eng. Life Sci..

[8] Ivshina I.B., Tyumina E.A., Bazhutin G.A., Vikhareva E.V. (2021). Response of *Rhodococcus cerastii* IEGM 1278 to toxic effects of ibuprofen. PLoS ONE.

[9] Blasco J., Trombini C. (2023). Ibuprofen and diclofenac in the marine environment—A critical review of their occurrence and potential risk for invertebrate species. Water Emerg. Contam. Nanoplastics.

[10] Jan-Roblero J., Cruz-Maya J.A. (2023). Ibuprofen: Toxicology and biodegradation of an emerging contaminant. Molecules.

[11] Sanchez-Aceves L., Perez-Alvarez I., Gomez-Olivan L.M., Islas-Flores H., Barcelo D. (2021). Long-term exposure to environmentally relevant concentrations of ibuprofen and aluminium alters oxidative stress status on *Danio rerio*. Comp. Biochem. Physiol. C.

[12] Marchlewicz A., Guzik U., Hupert-Kocurek K., Wojcieszyńska D. (2023). Evaluation of the defined bacterial consortium efficacy in the biodegradation of NSAIDs. Molecules.

[13] Najim A.A., Radeef A.Y., al-Doori I., Jabbar Z.H. (2024). Immobilization: The promising technique to protect and increase the efficiency of microorganisms to remove contaminants. J. Chem. Technol. Biotechnol..

[14] Dzionek A., Wojcieszyńska D., Marchlewicz A., Smułek W., Potocka I., Jałowiecki Ł., Borgulat J., Płaza G., Guzik U. (2024). Naproxen as environmental pollution, its effect on bacteria metabolism and degradation mechanism in immobilized *Planococcus* sp. S5. Chem. Eng. J..

[15] Marchlewicz A., Guzik U., Hupert-Kocurek K., Nowak A., Wilczyńska S., Wojcieszyńska D. (2017). Toxicity and biodegradation of ibuprofen by *Bacillus thuringiensis* B1(2015b). Environ. Sci. Pollut. Res..

[16] Dzionek A., Wojcieszyńska D., Adamczyk-Habrajska M., Guzik U. (2020). Enhanced degradation of naproxen by immobilization of *Bacillus thuringiensis* B1(2015b) on *Loofah* sponge. Molecules.

[17] Sutherland I.W. (2001). The biofilm matrix—An immobilized but dynamic microbial environment. Trends Microbiol..

[18] Bochner B.R., de Bruijn F.J. (2011). Phenomics and Phenotype Microarrays: Applications Complementing Metagenomics. Handbook of Molecular Microbial Ecology I: Metagenomics and Complementary Approaches.

[19] Marchlewicz A., Guzik U., Smułek W., Wojcieszyńska D. (2017). Exploring the degradation of ibuprofen by *Bacillus thuringiensis* B1(2015b): The new pathway and factors affecting degradation. Molecules.

[20] Otto M., Dickey S.W., Wolz C. (2023). Editorial: Quorum-sensing in gram-positive pathogens—Mechanisms, role in infection, and potential as a therapeutic target. Front. Cell. Infect. Microbiol..

[21] Prazdnova E.V., Gorovtsov a.v., Vasilchenko n.g., Kulikov M.P., Statsenko V.N., Bogdanova A.A., Refeld A.G., Brislavskiy Y.A., Chistyakov V.A., Chikindas M.L. (2022). Quorum-sensing inhibition by gram-positive bacteria. Microorganisms.

[22] Asma S.T., Imre K., Morar A., Herman V., Acaroz U., Mukhtar H., Arslan-Acaroz D., Shah S.R.A., Gerlach R. (2022). An overview of biofilm formation-combating strategies and mechanisms of action of antibiofilm agents. Life.

[23] Roy R., Tiwari M., Donelli G., Tiwari V. (2018). Strategies for combating bacterial biofilms: A focus on anti-biofilm agents and their mechanisms of action. Virulence.

[24] Ding Y., Liu H., Nanayakkara N.P.D., Khan I.A., Tekwani B.L., Walker L.A., Doerksen R.J. (2014). Hydroxylated derivatives of NPC1161: Theoretical insights into their potential toxicity and the feasibility and regioselectivity of their formation. J. Phys. Chem. A.

[25] Bhalla R., Tehrani R., Van Aken B. (2016). Toxicity of hydroxylated polychlorinated biphenyls (HO-PCBs) using the bioluminescent assay *Microtox*. Ecotoxicology.

[26] Dzionek A., Dzik J., Wojcieszyńska D., Guzik U. (2018). Fluorescein diacetate hydrolysis using the whole biofilm as a sensitive tool to evaluate the physiological state of immobilized bacterial cells. Catalysts.

[27] Mara K., Decorosi F., Viti C., Giovannetti L., Papaleo M.C., Maida I., Perrin E., Fondi M., Vaneechoutte M., Nemec A. (2012). Molecular and phenotypic characterization of *Acinetobacter* strains able to degrade diesel fuel. Res. Microbiol..

[28] Galardini M., Mengoni A., Biondi E.G., Semeraro R., Florio A., Bazzicalupo M., Benedetti A., Mocali S. (2014). DuctApe: A suite for the analysis and correlation of genomic and OmniLog Phenotype Microarray data. Genomics.

[29] Greń I., Wojcieszyńska D., Guzik U., Perkosz M., Hupert-Kocurek K. (2010). Enhanced biotransformation of mononitrophenols by *Stenotrophomonas maltophilia* KB2 in the presence of aromatic compounds of plant origin. World J. Microbiol. Biotechnol..

